# Cysticercosis: Hooked by a Hooklet on Fine Needle Aspiration Cytology—A Case Report

**DOI:** 10.1155/2013/315834

**Published:** 2013-08-19

**Authors:** Manav Sawhney, Shubhra Agarwal

**Affiliations:** Babu Jagjivan Ram Memorial Hospital, Jahangir Puri, Delhi 110033, India

## Abstract

Cysticercosis is a systemic parasitic disease caused by the larval form of cestode *T. solium*. It has a worldwide distribution and is potentially harmful with variable clinical manifestations. The patient most commonly presents with subcutaneous and muscle involvement in the form of nodular lesions. The other most commonly involved sites include eye, brain, bladder wall, and heart. Cysticercosis can be diagnosed on serology, and radiologically but confirmatory diagnosis is based on histopathological examination of the involved tissue biopsy specimen. Fine needle aspiration cytology is a useful low-cost outpatient procedure tool for preoperative diagnosis of cysticercosis and is absolutely essential for diagnosis of the parasitic lesions in a peripheral hospital, one like ours.

## 1. Introduction

Human cysticercosis is the larval infestation of the cestode *T. solium.* The diagnostic role of  FNAC in cysticercosis was first emphasized by Kung et al. in 1989 [1]. Since then FNAC has become a pivotal tool in evaluating subcutaneous and muscle nodules caused by parasites. Cysticercosis presents with wide spectrum of cytomorphological patterns ranging from viable cysts, cuticle fragments, scolex, and parenchyma to necrotic and calcified lesions. We report a case of cysticercosis diagnosed on FNAC emphasizing that the cytological diagnosis can be quite clear cut and without much ambiguity in cases where the actual parasite structure is identified in the smears [2]. It also at many times helps in avoiding unnecessary open biopsy for tissue diagnosis.

## 2. Case Report

**Figure 1:**
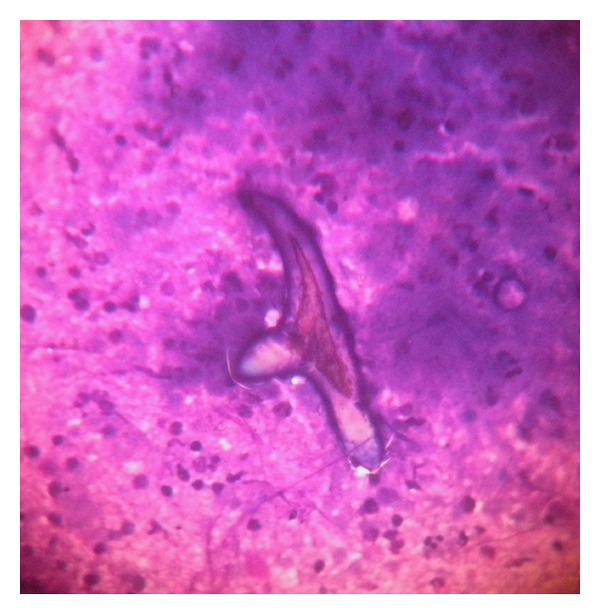
Showing hooklet of cysticercus in an inflammatory background.

A 4-year-old male patient presented with a superficial lump on left side of the abdominal wall since 1 month. The differential diagnosis of a lipoma, an abscess, or a tuberculous abscess was considered by the clinicians. The patient was sent to the pathology department for FNAC, it being the primary investigation in such cases. On examination, the lump measured 3.5 × 3 cm in size, slightly tender and soft to firm in consistency. Fine needle aspiration was done using 22G needle and 10 mL syringe without aspiration technique and yielded straw colored slightly turbid fluid. The smears were air dried and stained with May Grunwald Giemsa, haematoxylin, and eosin stains. Modified Ziehl Neelson stain was performed on one smear to rule out tuberculosis. On microscopic examination, fair number of neutrophils, lymphocytes, palisading histiocytes, eosinophils, and degenerated cells were noted. No granuloma or atypical cells were seen. Presence of eosinophils warranted closer look and on careful examination of five smears made from the aspirate, a solitary detached hooklet of cysticercus (Figure 1) was noted along with the inflammatory background. A diagnosis of cysticercosis cellulosae infection was made. The cytological diagnosis was further confirmed on serological examination for cysticercus. A final diagnosis of subcutaneous cysticercosis was given. The patient was advised antihelminthic therapy and antibiotics and is responding well. The patient also underwent imaging techniques, and lesions at other sites were ruled out.

## 3. Discussion

Human cysticercosis is the larval infestation of the cestode Taenia solium. Though cysticercus can be found in any organ, it is especially common in skeletal muscle and subcutaneous tissue as nodular lesions and is difficult to differentiate from benign mesenchymal tumors and lymphadenitis on clinical grounds alone [1]. 

Fine needle aspiration cytology (FNAC) in cysticercosis is low-cost outpatient procedure for preoperative diagnosis and may even obviate the need for open biopsy [2]. The cytological diagnosis is quite clear cut and undemanding in cases where actual parasite structure is identified in the smears. The cytomorphological identification of larvae in FNAC smears by different workers has widened the diagnostic utility of FNAC in skin nodules [3]. The diagnosis of cysticercus cellulosae infection is made when fragments of larval cuticle, parenchyma, or scolex are identified [4]. The viable cyst and the necrotic and calcified lesions all have distinctive cytomorphological patterns. The viable cyst yields clear fluid and shows fragments of  bladder wall in a clear acellular background. There is little or no inflammatory response as the parasites produce taeniastatin and other poorly defined molecules that interfere with the cellular immune response [5]. A careful search for hooklets is indicated wherever there is clear fluid aspiration with eosinophil prominence. Single and detached hooklets may be only recognizable remnants in aspirates of calcified cysts [6]. Aspirates of necrotic and degenerated lesions may contain fragments of  bladder wall, including calcareous corpuscles, detached single hooklets [7], and an infiltration of inflammatory cells, associated with the development of foreign body granulomas. The physical factors such as the firm nonexpansile nature of the host tissue may limit the growth of the parasite and initiate the host inflammatory response. The presence of eosinophils, neutrophils, palisading histiocytes, giant cells, and a typical granular dirty background in an aspirate from subcutaneous nodule should alert the cytopathologist of a parasitic infestation. Nonetheless, in still some cases of cysticercosis, none of these features may be present, and the inflammatory infiltrate may also be variable.

Cysticercosis cellulose is more common than usually thought. In endemic areas, cysticercosis cellulose should be included in the differential diagnosis of nodular lesions including echinococcus, granulomatous lesions, and soft tissue swelling. Various diagnostic modalities employed to detect cysticercosis apart from FNAC include radiology, serology, and pathological examination [7]. CT and MRI scans, though sensitive in diagnosing cysticercosis in cases where the parasite involves the CNS, are very expensive, and they provide only supportive diagnosis. Serological tests are useful if positive but cannot rule out the disease with negative results. False positivity is expected with the past parasitic infection or cross-reactivity with other helminths. FNAC has emerged as a widely acceptable method for the diagnosis of cysticercosis [7]. Thus, this case report helps to spread the awareness about the usefulness of fine needle aspiration cytology in the diagnosis of parasitic infestations and also emphasizes on careful search of all the smears in case of any inflammatory lesion.
